# Synergistic anti-biofilm effects of *Brassicaceae* plant extracts in combination with proteinase K against *Escherichia coli* O157:H7

**DOI:** 10.1038/s41598-020-77868-4

**Published:** 2020-12-03

**Authors:** Wen Si Hu, Da Min Nam, Joo-Sung Kim, Ok Kyung Koo

**Affiliations:** 1 Department of Food and Nutrition, Gyeongsang National University, Jinju, Republic of Korea; 2 Research Group of Consumer Safety, Korea Food Research Institute, Wanju-gun, Jeollabuk-do Republic of Korea; 3 Department of Food Biotechnology, Korea University of Science and Technology, Daejeon, Republic of Korea; 4 Institute of Agriculture and Life Science, Gyeongsang National University, Jinju, Republic of Korea

**Keywords:** Antimicrobials, Applied microbiology, Biofilms

## Abstract

Bacteria can form biofilms, complex microbial communities protected from environmental stress, on food contact surfaces. *Brassicaceae* plant has been shown to contain bioactive compounds with antimicrobial activities. The objective of this study was to evaluate the synergistic effects of *Brassicaceae* species and proteinase K against *E. coli* O157:H7 biofilm. We determined the minimum biofilm inhibitory concentration, the fractional inhibitory concentration indexes, and the synergistic inhibitory effect of *Raphanus sativus* var. *longipinnatus*, *R. sativus*, and *Brassica oleracea* var. *acephala* extracts with proteinase K on *E. coli* O157:H7. The biofilm showed a 49% reduction with 2 mg/mL *R. sativus*. The combination of proteinase K 25 µg/mL significantly increased the effect of 2 mg/mL *R. sativus* var. *longipinnatus* and the combined treatment yielded up to 2.68 log reduction on stainless steel coupons. The results showed that the combination of *R. sativus* var. *longipinnatus* extract and proteinase K could serve as an anti-biofilm agent with synergistic effects for inhibiting *E. coli* O157:H7 biofilm on stainless steel surfaces.

## Introduction

*Escherichia coli* O157:H7 is a major foodborne pathogen, which causes abdominal pain, diarrhea and even hemolytic uremic syndrome in humans worldwide^1^. A major source of *E. coli* O157:H7 is contaminated ground beef. However, fresh produce has also been recognized as an important source for the transmission of *E. coli* O157:H7 and has been implicated in an increasing number of foodborne outbreaks^2,3^. Fresh produce processing facilities can be involved in the transfer of contaminants through wash-water-mediated or direct contact with surfaces contaminated by a biofilm^4^. Without a sterilization step, it is nearly impossible to control the pathogen even with careful procedures.

Biofilms play an important role in cross-contamination by protecting pathogens from sanitary and sterilizing procedures^5^. Biofilms can form on any type of abiotic or biotic surface, resulting in serious problems in the food, marine, soil, and biomedical fields^6^. Many studies have tested the use of disinfectants, essential oils, plant extracts, and other chemicals to prevent biofilm formation or to remove existing biofilms^7^. Enzymes have been used for the treatment of biofilms formed in food areas^8^. Also, the application of enzymes on the cleaning of food contact surfaces has been approved by the regulatory agencies^9^. Proteinase K showed eradication of proteinaceous adhesins during the attachment step and disassembly of the extracellular polymeric substances^10^. Kim *et al*. used various enzymes, such as proteinase K and acylase I, for biofilm removal^10^. In another study, DNase I treatment was used for the removal of extracellular deoxyribonucleic acid, an architectural element of biofilms^11^. Traditional disinfectants are a health concern to consumers, and the extensive use of antibiotics causes an increase in resistant strains^12,13^. Plant-derived natural agents have been studied for their potential for biofilm reduction without any resistance or residue of toxic compounds. Recently, antibacterial properties have been studied in several natural compounds such as different plant extracts, essential oils^14^, and honey^15^. However, the sole use of natural agents alone has a limited antimicrobial activity, compared to the use of antibiotics and disinfectants. Therefore, in some studies, the combination of synthetic drugs with natural agents was investigated to enhance the antimicrobial effect^16,17^.

*Brassica* vegetables are highly nutritive and are recognized as providing antioxidative and antibacterial phytochemicals such as indole phytoalexins phenolics (feruloyl, isoferuloylcholine and hydroxybenzoic), and glucosinolates (glucoiberin, glucoraphanin and glucoalyssin). All of these phytochemicals contribute to the antioxidant, anticarcinogenic, and cardiovascular protective activities of *Brassica* vegetables^18^. In the *Brassicaceae* family, *Raphanus sativus* var. *longipinnatus* has been used in traditional medicine for disorders in the respiratory, urinary, and gastrointestinal systems^19^. *Brassicaceae* plant extracts, glucosinolate (GLS), and isothiocyanate (ITC) have been intensively investigated for their inhibitory effects on a wide range of microorganisms, including bacteria, fungi, insects, germinating seeds, and nematodes^20^. A previous study has shown that *Brassicaceae* plant extracts reduced *E. coli* O157:H7 during every step of biofilm formation^21^.

Hurdle technology combines various elements and processes to inhibit microbial growth^22^. Enterocin AS-48 treatment in combination with other agents such as EDTA, sodium tripolyphosphate, pH, and heat treatment in apple juice increased the permeability of planktonic *E. coli* O157:H7 cell membranes^23^. The use of antibiotics in combination with proteinase K showed stronger inhibitory effect on *Staphylococcus aureus* biofilm than a five-fold amount of gentamycin, streptomycin or ampicillin alone^24^. Kim *et al.* showed that combinatorial treatment of acylase I and proteinase K was more efficacious for eradicating *Pseudomonas aerugenosa* biofilm than either treatment alone^10^. Therefore, the purpose of this study was to evaluate the combinatorial effect of *Brassicaceae* plant extracts such as *R. sativus* var. *longipinnatus*, *R. sativus*, and *Brassica oleracea* var. *acephala* extracts with proteinase K on *E. coli* O157:H7 biofilm, and to evaluate their potential application to stainless steel surfaces.

## Materials and methods

### Bacteria strains and growth conditions

*E. coli* O157:H7 ATCC43894 was used from the culture collection of the Food Safety Laboratory at Gyeongsang National University. The bacterium was inoculated in a tryptic soy broth (TSB, Becton Dickinson Co., Franklin Lakes, NJ, USA) and incubated for 16–18 h at 37 °C in a shaking incubator. The cultures were maintained in 15% glycerol at − 80 °C until use.

### Preparation of plant extracts

All vegetables were purchased from grocery stores in Jinju-si (Republic of Korea). *R. sativus* var. *longipinnatus*, *R. sativus*, and *B. oleracea* var. *acephala* were freeze-dried in a freeze dryer (PVTFD50A, Ilsin Lab. Co., Gyeonggi-do, Republic of Korea). Next, a 25 g sample was suspended in 500 mL of 80% methanol and extracted at 30 °C in a shaking water bath at 100 rpm. The methanol extracts were evaporated at 30 °C. The extraction yield of each sample was from 9.04 to 47.5% (w/w).

### Biofilm formation assessment and crystal violet assay

Biofilm formation was assessed using crystal violet assay based on Lim et al. (2017)^25^. To initiate the attachment of bacteria, the culture medium was inoculated at approximately 10^7^ CFU/mL in polystyrene 96-well plates and incubated at 37 °C for 2 h under aerobic conditions. After the attachment step, the medium was carefully removed, and the 96-well plates were washed with PBS (phosphate-buffered saline, pH 7.0) to remove unattached cells. Fresh medium and the plant extracts and/or proteinase K were then added to evaluate the inhibitory effect on biofilm formation. Fresh media with and without *E. coli* O157:H7 were used as a positive and negative controls. After incubation at 37 °C for 18 h, a crystal violet (CV) assay was performed. The cultured well plate was washed with PBS to remove unattached cells. Then, 1% CV solution (bioWORLD, Dublin, OH, USA) was added, and the well plate was incubated for 30 min at room temperature. After the incubation, the well plate was washed three times with PBS and absolute ethanol was added and incubated for 15 min. After transferring the stained solution to a new well plate, the absorbance was measured at 595 nm using a SpectraMax M2 (Molecular Devices, Sunnyvale, CA, USA).

### Minimum biofilm inhibitory concentration (MBIC) assay

The MBIC was determined with three *Brassicaceae* plant extracts (*R. sativus* var. *longipinnatus*, *R. sativus*, and *B. oleracea* var. *acephala*) and proteinase K (P2308, Sigma-Aldrich, St. Louis, MO, USA). All assays were carried out in triplicate. This assay consisted of initial attachment at 37 °C for 2 h followed by the addition of one of the three plant extracts or proteinase K. After the initial attachment step, fresh media with and without *E. coli* O157:H7 were used as positive and negative controls. MBIC was determined as the lowest concentration of the extracts and proteinase K that resulted in complete inhibition of visible attachment in the CV assay.

### Checkerboard assay against *E. coli* O157:H7 biofilm

The synergistic antimicrobial effects of one of the three plant extracts (*R. sativus* var. *longipinnatus*, *R. sativus*, and *B. oleracea* var. *acephala*) in combination with proteinase K were assessed by a checkerboard assay based on Doern (2014)^26^. The results were analyzed using the fractional inhibitory concentration index (FICI). Briefly, the calculation of fractional inhibitory concentration (FIC) was compared with the value of MBIC of each agent alone and combined^24^. The sum of the FIC of each sample and the FIC of proteinase K was the FICI, as given in the following formula.

$${\text{FICI}} = \frac{{{\text{MBIC}}_{{\text{A}}} \;{\text{in}}\;{\text{combination}}}}{{{\text{MBIC}}_{{\text{A}}} \;{\text{alone}}}} + \frac{{{\text{MBIC}}_{{\text{B}}} \;{\text{in}}\;{\text{combination}}}}{{{\text{MBIC}}_{{\text{B}}} \;{\text{alone}}}}$$

MBIC_A_; the MBIC of treatment A, MBIC_B_; the MBIC of treatment B.

The FICI was determined using the following definition. An FICI of ≤ 0.5 was defined as synergistic, and an FICI in the range of 0.5–1 was defined as additive. An FICI of 1–4 was defined as indifferent, and an FICI of > 4 was defined as antagonism^27^.

### Biofilm formation assessment on stainless steel coupon

Stainless steel coupons (#304, 2 cm × 2 cm × 0.5 cm) were selected to examine the anti-biofilm effect, since most popular food-contact surfaces are made of stainless steel. The coupons were prepared as follows: immersion in alkaline detergent for 5 min, rinsing with distilled water (DW), and sonication for 10 min. After washing, the coupons were autoclaved for sterilization. The stainless steel coupons were placed in a 6-well plate, and 5 mL of the inoculum with approximately 10^7^ CFU/mL was added and incubated at 37 °C for 2 h to initiate attachment. After attachment, the culture medium was carefully removed, and the coupons were washed with PBS. Fresh medium with one of the *Brassicaceae* plant extracts and/or proteinase K was added and incubated for another 18 h under aerobic conditions to allow biofilm formation. The treatment concentrations were 1 mg/mL *R. sativus* var. *longipinnatus* extract and 100 µg/mL proteinase K, 2 mg/mL *R. sativus* extract and 100 µg/mL proteinase K, and 4 mg/mL *B. oleracea* var. *acephala* extract and 200 µg/mL proteinase K. After incubation, the coupons were washed with PBS and placed in a 50 mL tube containing 15 mL of PBS and 3 g of sterilized glass beads (5-mm diameter). Tubes were vortexed for 1 min and the suspended cells were serially diluted in TSB and spread on trypticase soy agar (TSA, Becton Dickinson Co.) plates for enumeration.

### Field emission-scanning electron microscopy (FE-SEM)

Field emission scanning electron microscopy was performed based on Lim and Kim (2017)^28^. The stainless steel was prepared with the treatment of *R. sativus* var. *longipinnatus* extract 1 mg/mL and proteinase K 100 µg/mL. After treatment, the samples were fixed with Karnovsky's glutaraldehyde solution containing 0.05 M sodium cacodylate buffer (Sigma-Aldrich), 2% paraformaldehyde (T&I, Gangwon, Republic of Korea), and 2% glutaraldehyde (Georgia Chem, Norcross, GA, USA) at 4 °C for 2 h. After washing twice with 0.05 M sodium cacodylate buffer twice, the samples were incubated in 1% osmium tetroxide (Sigma-Aldrich) with 0.05 M cacodylate buffer at 4 °C for 2 h. The samples were then washed in distilled water, and dehydrated with increasing alcohol concentrations (30%, 50%, 70%, 80%, 90% and 100%). The samples were dried with hexamethyldisilazane (Sigma-Aldrich) for 18–24 h in a biosafety cabinet, and then the samples were sputter-coated with gold (JFC 1100E ion sputtering device, EG&G, USA). The gold-coated samples were examined with a FE-SEM (Philips XL30S FEG, Philips, Eindhoven, Netherlands).

### Statistical analysis

Statistical significance was determined by Duncan's multiple range test and a Student-t test procedure of SPSS 12.0 (SPSS Inc., Chicago, IL, USA). The level of statistical significance was *p* < 0.05.

## Results and discussion

### Minimum biofilm inhibitory concentration of plant extracts and proteinase K

*Brassicaceae* plant extracts and proteinase K were tested for their inhibitory effect on *E. coli* O157:H7 biofilm. In this study, *E. coli* O157:H7 ATCC43894 (CDC EDL 932) was selected to represent the contamination of pathogenic *E. coli* on surfaces. This strain is one of the most important foodborne pathogens worldwide, and showed the ability to attach and form biofilms on a variety of food surfaces. This strain has been used as a representative strain for numerous biofilm formation related studies^21,25,29,30^. Accordingly, this strain can be a practical significance to study *E. coli* O157:H7 and other EHEC serogroups.

Anti-biofilm effect was tested after the 2 h of initial attachment step. This condition was selected from the previous study with the evaluation of inhibition effect on each step of biofilm formation process; treatment during initial attachment step (anti-attachment), treatment during biofilm development (anti-biofilm), and treatment after biofilm establishment (post anti-biofilm)^21^. Since the extracts were the most effective during the biofilm development, the minimum biofilm inhibitory concentration (MBIC) was evaluated by treatment after the initial attachment stage of biofilm formation. MBIC values of *R. sativus* var. *longipinnatus*, *R. sativus*, and *B. oleracea* var. *acephala* extracts were compared (Table 1). When testing proteinase K, the MBIC value was 1000 µg/mL. *E. coli* O157:H7 biofilm was sensitive to *R. sativus* var. *longipinnatus*, *R. sativus*, and *B. oleracea* var. *acephala* extracts with MBIC of 4, 4, and 8 mg/mL respectively.

**Table 1:** Minimum biofilm inhibitory concentration (MBIC) and fractional inhibitory concentration (FIC) of *R. sativus* var. *longipinnatus*, *R. sativus*, and *B. oleracea* var. *acephala* extracts with proteinase K against *E. coli* O157:H7.

	Concentration of plant extract (mg/mL)			Concentration of proteinase K (µg/mL)			FICI^§^
	MBIC of plant extract only	MBIC of plant extract with proteinase K	FIC* of plant extract	MBIC of proteinase K only	MBIC of proteinase K with plant extract	FIC proteinase K	
*R. sativus* var. *longipinnatus*	4	1	0.25	1000	100	0.1	0.35
*R. sativus*	4	2	0.5	1000	100	0.1	0.6
*B. oleracea* var. *acephala*	8	4	0.5	1000	200	0.2	0.7

*Fractional inhibitory concentration (FIC) was calculated from the MBIC of the combined agents divided by the MBIC of each agent alone.

^§^Fractional inhibitory concentration index (FICI) is the sum of the FIC of each extract and the FIC of proteinase K. The calculation was based on Odds (2003)^25^.

In a previous study, *Brassicaceae* plant extracts required an average concentration of 4 mg/mL or more to control *E. coli* O157:H7 biofilm formation^21^. The mechanism of reduction by the *Brassicaceae* plant extracts is unknown but some active compounds, such as caffeic acid, gallic acid and isothiocyanates (ITC), reduced the *E. coli* O157:H7 biofilm^21^. Lu et al. (2016) showed that *Wasabia japonica* (Japanese horseradish) extract containing 59 µg/mL of allyl ITC inhibited *E. coli* growth^31^. Studies on 2-thioxo-3-pyrrolidinecarbaldehyde in *R. sativus* var. *longipinnatus* documented antimicrobial activity, with the minimum inhibitory concentration against bacteria ranging from 50 to 400 µg/mL^32,33^. The results showed the removal of bacteria on biofilm formation rather than the bactericidal activity with the natural vegetable extracts.

### Synergistic inhibition by plant extracts and proteinase K

**Figure 1:**
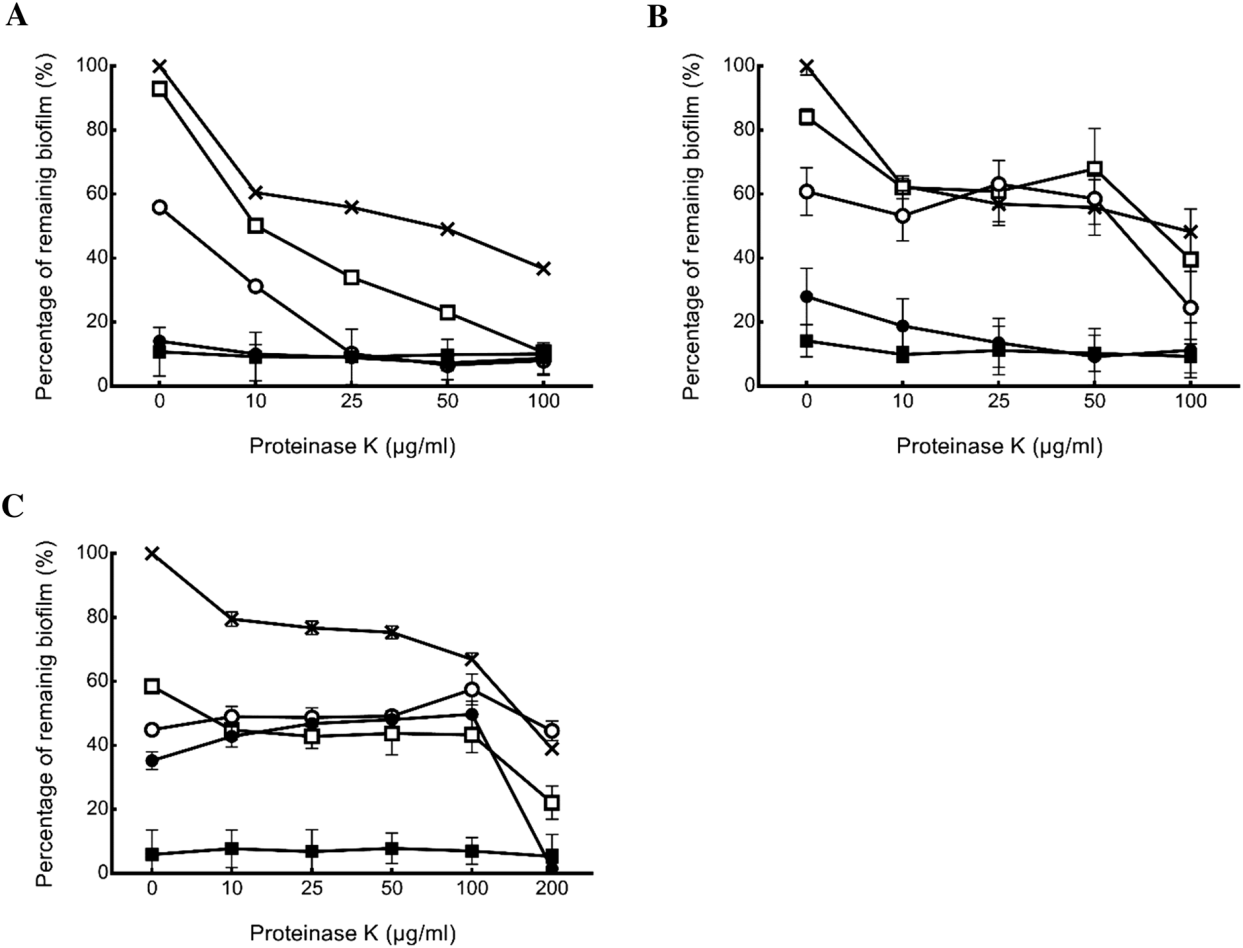
The effect of *Brassicaceae* plant extracts combined with proteinase K on *E. coli* O157:H7 biofilms. Percentage of biofilm remaining compared to the biofilm without any treatment after proteinase K treatment with *R. sativus* var. *longipinnatus* (**A**), *R. sativus* (**B**) and *B. oleracea* var. *acephala* (**C**) extracts. Concentration of extracts were 8 mg/mL (■), 4 mg/mL (●), 2 mg/mL (○), 1 mg/mL (□) and 0 mg/mL (×). Error bars represent standard error.

The inhibitory effect of the combination of *Brassicaceae* plant extracts and proteinase K on *E. coli* O157:H7 biofilm was analyzed using a checkerboard assay. In the combinatorial treatments, *Brassicaceae* plant extracts at 1–8 mg/mL and proteinase K at 10–200 µg/mL were used, and the results are expressed as dose–response curves (Fig. 1). Treatment with 1 mg/mL *R. sativus* var. *longipinnatus* extract showed the reduction of the biofilm from 7% (with *R. sativus* var. *longipinnatus* extract alone) to 89% (*R. sativus* var. *longipinnatus* extract combination with proteinase K of 100 µg/mL) (Fig. 1A). At the same concentration, *R. sativus* extract resulted biofilm reduction from 17% with *R. sativus* extract alone to 60% in combination with proteinase K (Fig. 1B). When the *B. oleracea* var. *acephala* extract was used, the biofilm reduction ranged from 41 to 65% at concentrations of 1 to 4 mg/mL. Biofilm was significantly reduced from 65 to 98%, when 4 mg/mL *B. oleracea* var. *acephala* extract and 200 µg/mL proteinase K were treated together (Fig. 1C). The FIC index is presented in Table 1. When *R. sativus* var. *longipinnatus* extract and proteinase K were provided together, the MBIC of *R. sativus* var. *longipinnatus* extract decreased from 4 to 1 mg/mL. In addition, the combined treatment reduced the treatment concentration of *R. sativus* and *B. oleracea* var. *acephala* extracts from 4 to 2 mg/mL and 8 to 4 mg/mL, respectively. Furthermore, the MBIC value of proteinase K showed a decrease from 1000 to 100 µg/mL when combined with *R. sativus* var. *longipinnatus* extract. *R. sativus* var. *longipinnatus* had a synergistic effect (FIC index < 0.5) with proteinase K with an FICI value of 0.25. This method can enhance the efficiency of biofilm formation inhibition. *R. sativus* var. *longipinnatus* extracts have been studied as a potentially effective anti-biofilm agent in the biofilm development stage^21^.

Proteinase K is a serine protease, which cleaves peptide bonds C-terminal to breaks down the aliphatic and aromatic amino acids for protein digestion^34^. The major components of *E. coil* biofilms are the extracellular polymeric substances (EPS) including nucleic acids, lipids, proteins and exopolysaccharides, which take up to 90% of the dry weight of the biofilm^35^. Curli, an extracellular protein fiber, is present in the EPS of *E. coli* and it was degraded by proteinase K to reduce the biofilm formation^36^. However, proteinase K did not reduce the growth rate of *E. coli* O157:H7^37^. Former researchers used proteinase K against a wide range of biofilm forming bacteria for food processing facilities and directly on food. Proteinase K were effective to inhibit *Listeria monocytogenes* and *Staphylococcus aureus* biofilms on polystyrene and *E. coli* O157 biofilm on cucumber^7,24,38^. Properly rinsed surfaces has no possibility of food contamination nor the risk for any enzyme to be considered pollutants^39^. Based on aforementioned studies and our study, the proteinase K was able to reduce biofilm and to show the prospective applications in food industry. When proteinase K and *R. sativus* var. *longipinnatus* extract are combined, proteinase K may decompose related proteins at the time of initial attachment, thereby readily causing the exposure of the sessile cells to the *R. sativus* var. *longipinnatus* extract and broadening the antimicrobial range of *R. sativus* var. *longipinnatus* extract. Proteinase K and natural substances (thyme oil liposomes) have also shown synergistic effects on inhibiting biofilm formation^38^. Therefore, the combined treatment using proteinase K and *R. sativus* var. *longipinnatus* extract appears to be synergistic by affecting biofilm attachment and development.

### Inhibition of biofilm formation on stainless steel surfaces

**Figure 2:**
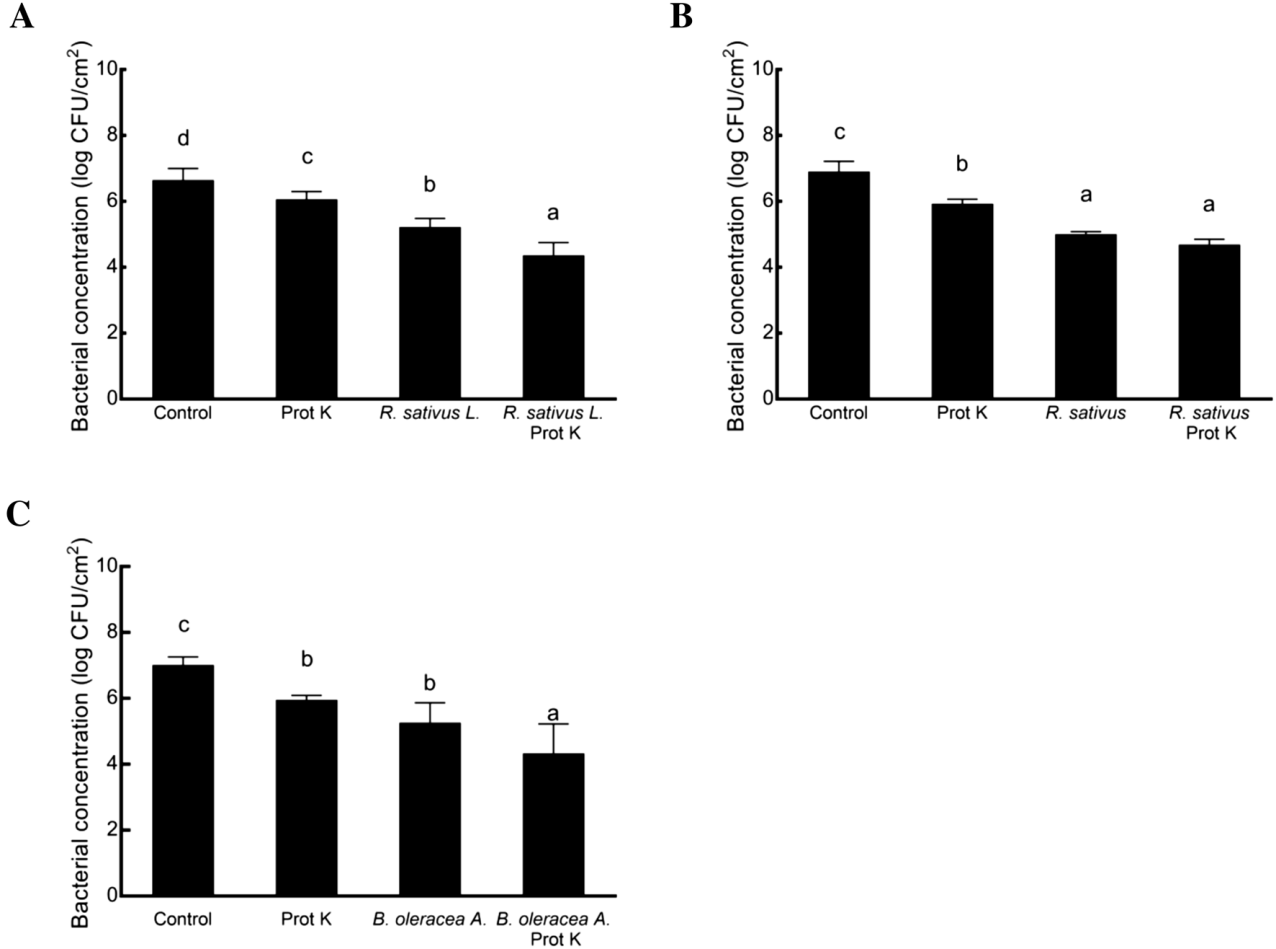
Inhibition of *E. coli* O157:H7 biofilm formation by proteinase K (prot K) and *R. sativus* var. *longipinnatus* (**A**), *R. sativus* (**B**) and *B. oleracea* var. *acephala* (**C**) extracts on stainless steel coupons. The treated concentrations of the plant extracts and proteinase K, were 1 mg/mL *R. sativus* var. *longipinnatus* extract and 100 µg/mL proteinase K, 2 mg/mL *R. sativus* extract and 100 µg/mL proteinase K, and 4 mg/mL *B. oleracea* var. *acephala* extract and 200 µg/mL proteinase K. The control is biofilm formation of *E. coli* O157:H7 on stainless steel without any treatment. Error bars represent standard error. Different lowercase letters are significantly different (*P* ˂ 0.05).

To examine the combination effect on food-contact surfaces, we compared the survival rates of *E. coli* O157:H7 on stainless steel surfaces. In this study, the results were determined by assessing the viable cell count (Fig. 2). The *E. coli* O157:H7 living cells in biofilm decreased from 6.66 log CFU/cm^2^ to 6.07, 5.23, and 4.37 log CFU/cm^2^ with proteinase K, *R. sativus* var. *longipinnatus* extract, and a combination thereof, respectively. The reduction of the combined proteinase K and *R. sativus* var. *longipinnatus* extract was 2.29 log CFU/cm^2^, which was greater than the sum of proteinase K alone and *R. sativus* var. *longipinnatus* extract alone. In this experiment, the combined treatments with proteinase K had significantly decreases in biofilm formation compared to the *Brassicaceae* plant extracts alone, with reductions of 2.29, 2.22, and 2.68 log CFU/cm^2^ for *R. sativus* var. *longipinnatus*, *R. sativus*, and *B. oleracea* var. *acephala* extracts, respectively. Overall, significant reduction was showed in all treatment group compared to the control. While the natural plant extract has certain antibacterial activity, it is hard to expect complete or close to complete removal as antibiotics. Our research aim was to discover and evaluate the combination of biofilm inhibitors on the cleaning and protection of surfaces to provide an efficiently greenery alternative to substitute the harmful and ineffective chemical biocides. Studies on the removal of bacteria from stainless steel surfaces using disinfectants are important for solving hygiene problems in the food industry. It has been reported that the biofilm of *E. coli* O157:H7 is more resistant to disinfectants than planktonic cells on food-contact surfaces^40^. While essential oils can be an alternative to disinfectants, they require high concentrations with a strong organoleptic flavor to affect the taste of food products^41^. Therefore, the combination of proteinase K and *Brassicaceae* plant extracts may be a possible alternative antimicrobial agent on stainless steel surfaces.

### Field emission-scanning electron microscopy (FE-SEM)

**Figure 3:**
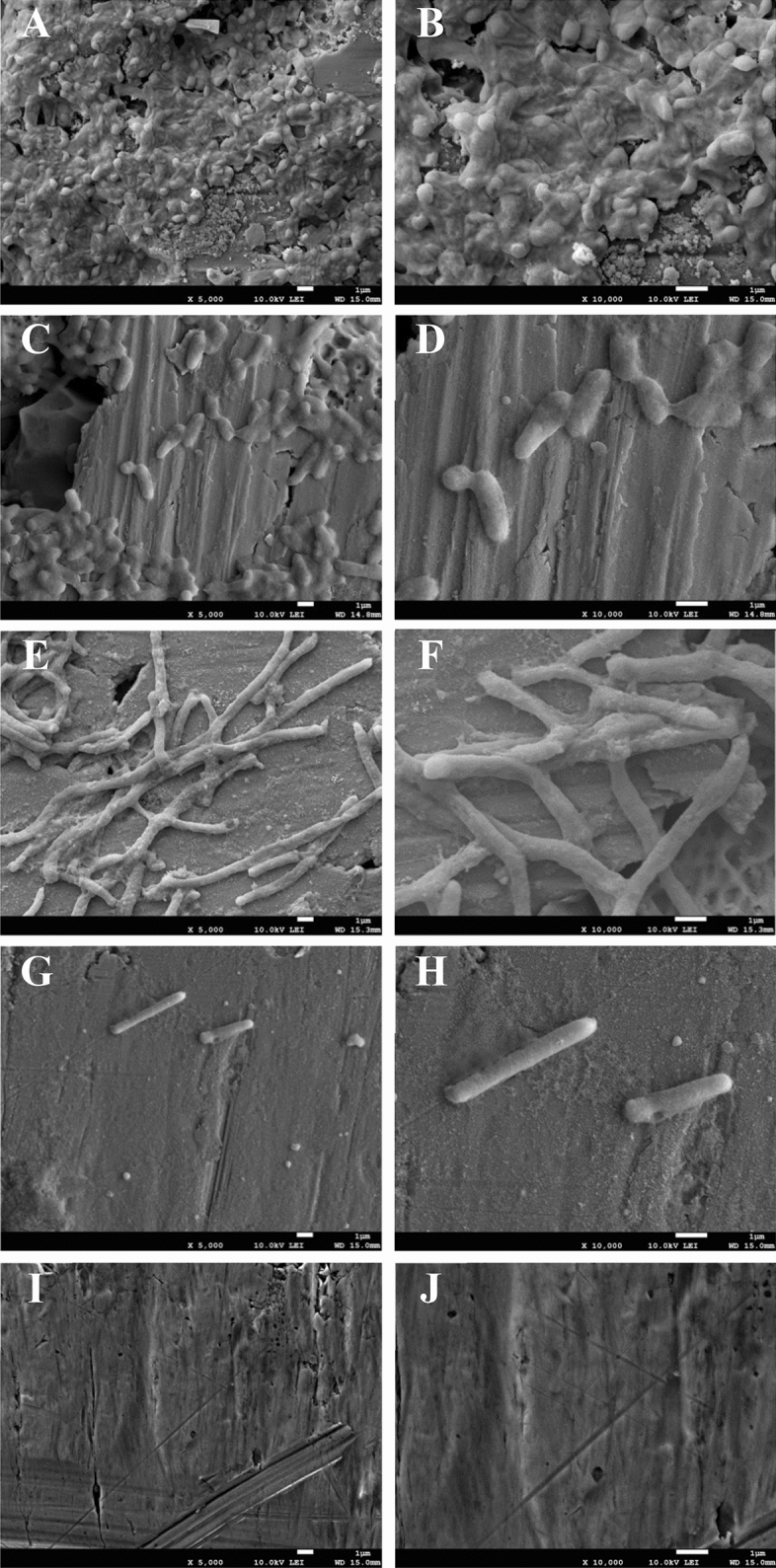
Field-emission scanning electron microscopy analysis of biofilm eradication on stainless steel coupon. Biofilms formed by *E. coli* O157:H7 were exposed to 100 µg/mL proteinase K (**C**,**D**) and, 1 mg/mL *R. sativus* var. *longipinnatus* extract (**E**,**F**) alone or the combination thereof (**G**,**H**) and compared with a positive control (**A**,**B**). Negative controls (**I**,**J**) are an untreated stainless steel coupon. The magnification of **A**,**C**,**E**,**G** and, I is 5000×, and **B**,**D**,**F**,**H** and, **J** is 10,000x.

The treated bacteria were observed using FE-SEM to investigate the morphology of bacteria in response to the combined treatment on the stainless steel surface (Fig. 3I and J). *R. sativus* var. *longipinnatus* extract at 1 mg/mL and proteinase K at 100 µg/mL were used based on the FICI index (Table 1). Bacterial cells were embedded in a biofilm layer with the production of an extracellular matrix (Fig. 3A and B). Both proteinase K (Fig. 3C and D) and *R. sativus* var. *longipinnatus* extract (Fig. 3E and F) decreased the number of bacterial cells and reduced the development of extracellular polymeric substances. Interestingly, *R. sativus* var. *longipinnatus* extract hindered the bacterial cell division*,* producing elongated *E. coli* O157:H7, while proteinase K only reduced the number of attached cells. Bacterial cells treated with the combination of *R. sativus* var. *longipinnatus* extract and proteinase K were markedly decreased with only elongated cells remaining, and most of the outermost layer of the bacterial cells disappeared (Fig. 3G and H). Further, they showed the synergistic effect of proteinase K treatment when used with antibiotics.

Inhibition of bacteria by *R. sativus* var. *longipinnatus* extract was mediated through cell division inhibition, of which can be caused by targeting the related genes. Several research groups studied the inhibitory effect of berberine and curcumin on the cell division related genes, with the disruption of the biofilms of *E. coli,* and *S. aureus, S. epidermidis,* and *Enterococcus*, respectively, by inhibiting the assembly of FtsZ in the Z-ring^42,43^. The bacterial cell division and septum formation play a vital role in the process of the formation and development of biofilm**.** This type of treatment can lead to bacteriostatic control during treatment. While further study on the inhibition mechanism of the extracts is needed, our study shows a potential action on cell division.

In conclusion, the combination of *R. sativus* var. *longipinnatus* extract and proteinase K has synergistic activity against *E. coli* O157:H7 biofilms. The synergistic effect of *R. sativus* var. *longipinnatus* extract and proteinase K may result from the disruption of the biofilm structure and inhibition of biofilm persistence by interrupting cell division with the assembly of cell division proteins. The results from this study support the potential use of *R. sativus* var. *longipinnatus* extract to control *E. coli* O157:H7 in combination with other methods.
